# Influenza-Associated Hospitalization in a Subtropical City

**DOI:** 10.1371/journal.pmed.0030163

**Published:** 2006-03-07

Influenza is estimated to be responsible for a million deaths worldwide every year. In developed countries, epidemics of influenza have long been known to increase hospital admissions and mortality. This impact has been measured quantitatively in many studies dating back over a century. The statistical methods used in such research have involved both “comparative” and, more recently, “regressive” techniques.

There are of course many difficulties in collecting and analyzing this kind of information in developing countries. In addition, the lack of clear seasonal patterns to epidemics in these regions prevents the use of regressive statistical methods. As a result, the impact of influenza on morbidity and hospitalization in the tropics and subtropics has been poorly quantified. However, the Hong Kong Special Administrative Region, which has a subtropical climate and a modern health-care system, presents opportunities to conduct such research. Ninety-five percent of the people admitted to hospital in Hong Kong go to public-sector hospitals, which have a central computerized system in which clinical records are kept on all patients.

In a new study, Peiris and colleagues present the results of a study in which they obtained information on patients admitted to hospitals in Hong Kong during the period 1996–2000. They developed an appropriate statistical technique that allowed them to capture influenza-associated mortality even in the absence of a predictable seasonal pattern of influenza, and also controlled for potential confounding factors as variations in temperature and humidity. The researchers found that during influenza outbreaks, hospital admissions increased, not just for respiratory diseases such as pneumonia but also for cardiovascular conditions and diabetes. The increases were most noticeable for older people. Overall, influenza was responsible for 11.6% of admissions for respiratory disease, 1.5% of admissions for stroke, 1.8% of admissions for heart attacks, and 3.5% of admissions for diabetes. These figures are comparable with what has been found in developed countries outside the tropics.

The finding that the influenza burden faced by Hong Kong is in fact similar to that in the United States, for example, is important, as it has usually been assumed that influenza does not have a significant impact on health outside the temperate regions. The results of this study suggest that influenza deserves to be given a higher priority than it is accorded at present in tropical and subtropical countries. The authors urge the introduction of vaccination programs for people at high risk, particularly the elderly.

Given the special circumstances of Hong Kong, some caution is of course required when extrapolating these findings to other parts of the tropics and subtropics. A wealthy subtropical city with good infrastructure is different in very many respects from low-income nations in the tropics. Most tropical countries face a massive disease burden from other medical conditions and lack the level of resources for health care that are available in Hong Kong. Nevertheless, this is clearly an important study. Its implications are discussed further in the Perspective by Viboud, Alonso, and Simonsen (DOI: 10.1371/journal.pmed.0030089). They note, in particular, the finding that hospitalization of children with influenza is apparently greater in Hong Kong than in the US, and they suggest that childhood vaccination programs may need to be considered.

## 

**Figure:**
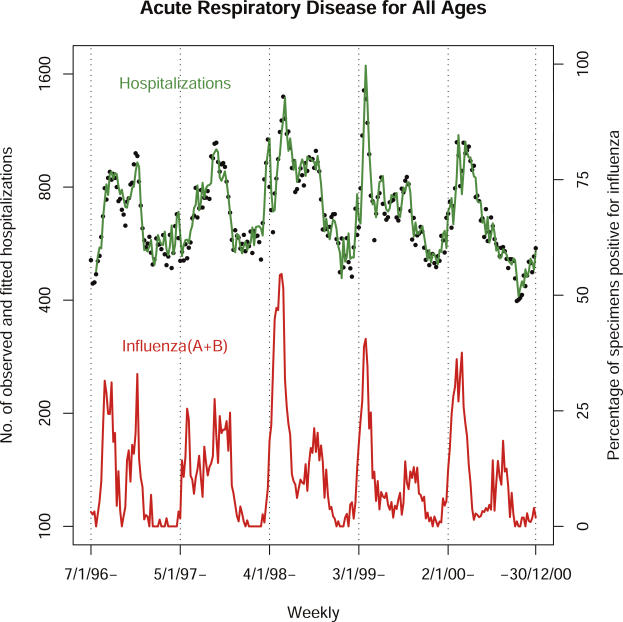
Weekly number of observed and fitted hospitalizations, and percentage of specimens positive for influenza

